# Photocatalytic Activity of TiO_2_-Doped Fe, Ag, and Ni with N under Visible Light Irradiation

**DOI:** 10.3390/gels8010014

**Published:** 2021-12-24

**Authors:** Byung-Geon Park

**Affiliations:** Department of Food and Nutrition, Kwangju Women’s University, 165 Sanjung-dong, Gwangju 62396, Korea; bgpark@kwu.ac.kr; Tel.: +82-62-950-0814

**Keywords:** TiO_2_ doped metal ions, nitrogen doping, visible light photocatalyst, formaldehyde decomposition, *Pseudomonas aeruginosa*

## Abstract

Doping with noble metal ions or doping with nitrogen has been attempted to prepare TiO_2_ that reacts even in visible light. In this study, TiO_2_ was doped with nitrogen and various metal ions instead of noble metals. The TiO_2_ photocatalysts doped with metal ions (Fe, Ag, Ni) and nitrogen were prepared by a sol-gel method. Their physicochemical properties were characterized and their photocatalytic activities were investigated under visible light irradiation. In TiO_2_ doped with metal ions and nitrogen, the light absorption region was extended to visible light. The photoluminescence intensity was much greater in N/Ni/TiO_2_ than in N/Ag/TiO_2_ and N/Fe/TiO_2_. The photolysis activities of N/Ni/TiO_2_ were the highest in formaldehyde decomposition and methylene blue decomposition. The sterilization efficiency of N/Ni/TiO_2_ was the highest in the evaluation test for the inhibition of the proliferation of *Pseudomonas aeruginosa*. The bandgap of N/Ni/TiO_2_ was 2.4 eV, which was significantly lower than that of anatase TiO_2_ (3.2 eV). The N/Ni/TiO_2_ had a much higher optical intensity than other metal ion-doped TiO_2_, so it was highly active under visible light irradiation.

## 1. Introduction

TiO_2_ is a representative photocatalyst due to its superb photocatalytic activities and excellent chemical stability [1]. TiO_2_ shows various photocatalytic properties. In particular, it is effective in decomposing organic pollutants as it has a strong oxidizing ability [2,3]. TiO_2_ has been applied in various fields due to its low cost and eco-friendly properties [4]. However, TiO_2_ has a large bandgap energy (3.0–3.2 eV), so the photocatalytic activity does not show for visible light, but only for UV light. In addition, there is a disadvantage in that the efficiency decreases due to rapid recombination [5,6].

To make the photocatalytic activity of TiO_2_ appear even in visible rays, studies are being actively attempted to reduce the bandgap by modifying the surface of TiO_2_ [7,8,9,10]. Doping metal ions onto the surface of TiO_2_ narrows the bandgap, which not only allows energy absorption to be extended to visible light but also improves charge separation and photocatalytic activity [11,12,13]. The visible light sensitization effects have been studied on TiO_2_ doped with various metals such as rhodium, palladium, platinum, gold, silver, ruthenium, cobalt, copper, and nickel [14,15,16,17]. Various photocatalytic activities were shown according to metal doping. The activities of metal-doped TiO_2_ are dependent on the properties of the TiO_2_ host, the type of metal, and the metal deposition sequence [18]. In addition, it has been reported that the bimetal photocatalyst shows excellent performance in H_2_ production because the metal acts as an electron sink for photoexcitation electrons [19,20,21].

The photochemical reaction occurs while generating electron and hole pairs by irradiating the photocatalyst with a light source that has energy corresponding to the bandgap of the photocatalyst. Electron/hole pairs generated by photocatalytic activity play an essential role in H_2_ production by water decomposition [22]. The photocatalytic activity of TiO_2_ is significantly affected by the bandgap and the light source [23]. The bandgap becomes wider, the wavelength of light absorption becomes narrower to the UV region, and when the bandgap is narrowed, the light absorption region is extended to the visible light region [24,25]. Doping TiO_2_ with metal acts as an electron trap, narrowing the bandgap, increasing TiO_2_ activity, and inhibiting electron–hole recombination [26]. Metal doping on TiO_2_ in the UV light system causes the Fermi level of the metal to be lower than that of the conduction band (CB) in TiO_2_ [27]. For noble metals, surface plasmon resonance (SPR) effects also occur [28,29]. The results show that metal doping can enhance photocatalytic activity and reduce recombination.

The doping of transition metal ions or rare earth metal ions on TiO_2_ can also enhance their photocatalytic activity. The doping of metal ions extends the photoreaction of TiO_2_ to the visible light area. As metal ions enter the lattice of TiO_2_, the energy levels of impurities are formed in the bandgap of TiO_2_. When metal ions are doped on the TiO_2_ surface, electrons/holes are difficult to transfer to the interface, so the metal ions tend to play a central role in recombination. However, there is an optimal value for the concentration of doped metal ions, and if doped at a higher concentration than this, recombination increases and the activity of the photocatalyst decreases [30].

Doping metals on TiO_2_ is also aimed at improving the transport of photo-generated electrons, which prolongs the lifetime of charge carriers. Usually, the photoactivity of metal-doped TiO_2_ is different depending on the type of dopant. Overall, When the TiO_2_ support was doped with Au, the activity was much higher than that of the Ag-doped TiO_2_ [31,32]. In this way, when TiO_2_ is doped with noble metals such as Au and Pt, the photocatalytic activity is much better than that of other metals. However, precious metals are expensive, which increases the production cost of the catalyst. In order to use a metal with a low price and high photocatalytic activity as a dopant, it is necessary to investigate the photocatalytic activity and physicochemical properties by doping various metals on TiO_2_.

It is known that when metal cations are doped, doped metal ions play a central role in electron–hole recombination, in which electrons that have moved to CB return to holes. That is, the recombination of electrons–holes is promoted due to metal doping, thereby reducing the reaction by electrons of CB or holes of the valance band (VB) [33,34]. On the other hand, the nitrogen doping effect is known to control the central role, along with the report that it promotes electron–hole recombination along with the generation of oxygen deficiency in the titania lattice structure [35]. Nitrogen doping is classified as anion doping during photocatalytic surface modification, along with carbon, sulfur, and fluorine doping. The advantage of anion doping is that the bandgap of the photocatalyst is narrowed, so that electron movement and hole generation occur even when visible light is irradiated, resulting in a photochemical reaction [36,37]. That is, anion doping serves to lower the bandgap energy, like cation doping. Nitrogen used for anion doping is known to have relatively superior efficacy compared to other anions in terms of photocatalytic performance [38].

In this study, various metal ions were doped on the surface of TiO_2_ using the sol-gel method so that TiO_2_ could exhibit photocatalytic performance even in visible light. The same amount of nitrogen was doped to each photocatalyst after doping with metal ions to maximize each photocatalyst’s activity. The physicochemical properties and photocatalytic activity of each TiO_2_ photocatalyst, according to the type of doped metal ions, were investigated. The photocatalytic activity of the metal-doped TiO_2_ photocatalysts was evaluated through the decomposition reaction of methylene blue (MB) and the decomposition reaction of formaldehyde (HCHO) under visible light irradiation. In addition, the bactericidal power of each photocatalyst was compared and evaluated through an experiment to inhibit the proliferation of *Pseudomonas aeruginosa*.

## 2. Results and Discussion

### 2.1. Physicochemical Properties of TiO_2_ Doped with Metal Ions and Nitrogen

**Figure 1:**
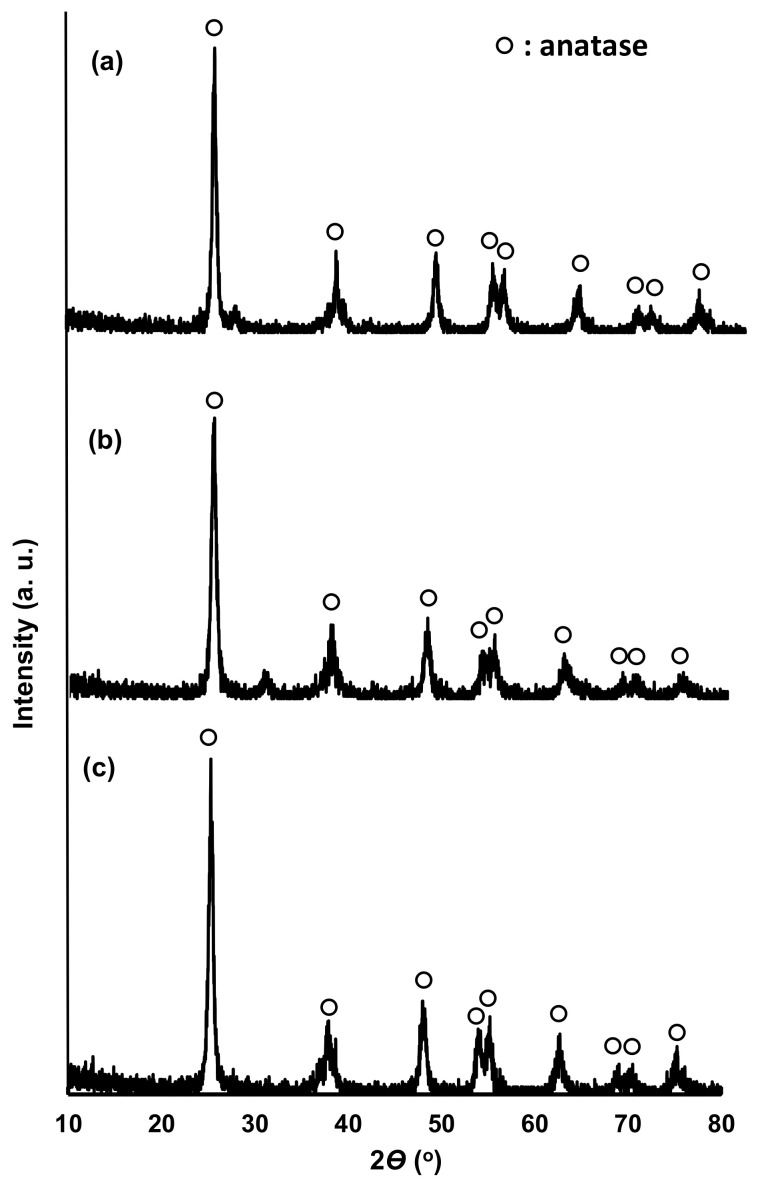
XRD patterns of (**a**) NFT, (**b**) NAT, and (**c**) NNT.

**Figure 2:**
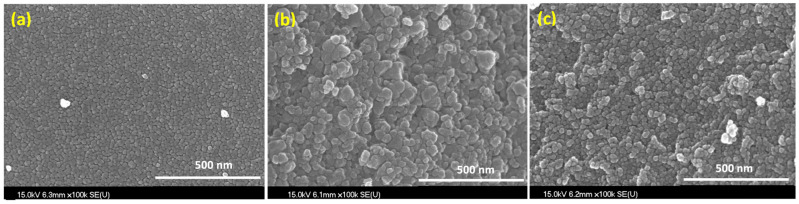
SEM images of (**a**) NFT, (**b**) NAT, and (**c**) NNT.

**Figure 3:**
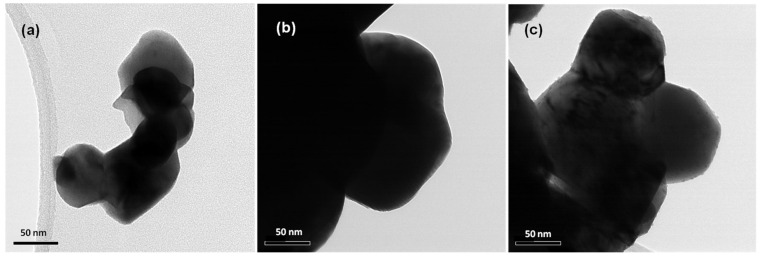
TEM images of (**a**) NFT, (**b**) NAT, and (**c**) NNT.

Figure 1 shows the X-ray diffraction (XRD) patterns of N/Fe/TiO_2_ (NFT), N/Ag/TiO_2_ (NAT), and N/Ni/TiO_2_ (NNT). The 2*Ɵ* positions of the peaks of the XRD patterns of the three photocatalysts were almost identical. The XRD patterns of the photocatalysts were almost identical to the typical XRD pattern of anatase TiO_2_ (JCPDP 27-1000) [39]. No peak of doped metal was observed. It seems that this has not been analyzed because the amount of doped metal after TiO_2_ preparation is very small (1 wt% or less). Figure 2 represents the scanning emission microscope (SEM) images of the photocatalysts. The crystallite sizes of NFT were smaller than those of NAT and NNT. The crystal shapes of NAT and NNT were irregular or nearly spherical. Figure 3 shows a transmission electron microscope (TEM) image of the photocatalyst. The crystallite size of NFT was smaller than that of other photocatalysts. The average crystal sizes of NNT and NAT, estimated from many TEM images, were about 70 nm. On the other hand, that of the NFT was slightly smaller, about 50 nm. The crystal morphology of the photocatalyst showed an irregular shape but was close to a spherical shape.

**Figure 4:**
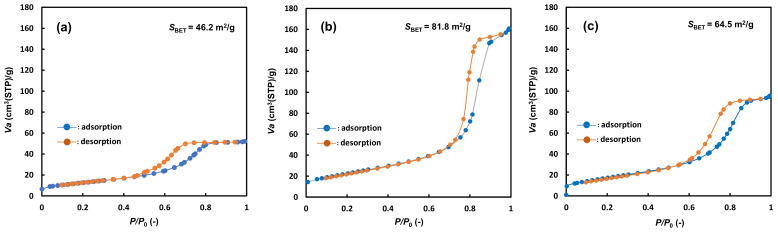
N_2_ isotherms of (**a**) NFT, (**b**) NAT, and (**c**) NNT.

Figure 4 shows the N_2_ adsorption isotherms of the photocatalysts. The adsorption isotherm of each photocatalyst was shown as a hysteresis curve. The BET [40] surface area obtained from the N_2_ isotherm was 46.2 m^2^/g for NFT, 81.8 m^2^/g for NAT, and 64.6 m^2^/g for NNT. The surface area of commercial TiO_2_ (Evonik, P25), used as a standard material, is known to be about 50 m^2^/g. The surface area of NFT was slightly smaller than that of TiO_2_ (P25). The surface areas of NNT and NAT were larger than that of TiO_2_ (P25). In particular, the surface area of NAT was increased significantly. These results mean that the crystal size of the photocatalyst prepared by the sol-gel method is small and uniform for TiO_2_ (P25), and thus the surface area is also large.

**Figure 5:**
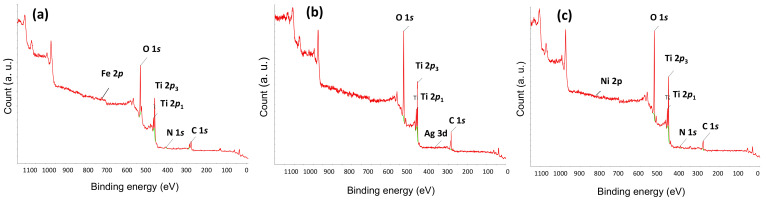
XPS spectra of (**a**) NFT, (**b**) NAT, and (**c**) NNT.

Figure 5 presents the X-ray photoelectron spectroscopy (XPS) results of the photocatalysts. In XPS, a Ti 2*p*_3_ peak at 459 nm and a Ti 2*P*_1_ peak at 464 nm appeared. The N 1*s* peak at 400 nm appeared by N doping. NFT showed a trace of the Fe 2*p* peak at 725 nm. The Ag 3*d* peak was observed at 372 nm for NAT. The Ni 2*p* peak at 870 nm was observed in the XPS results of NNT. The reason that the peaks of Fe, Ag, and Ni metals appear as traces in XPS results is that the amount of doped metal is very small.

**Figure 6:**
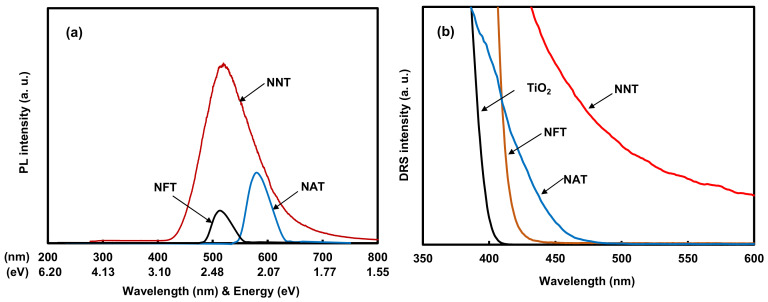
(**a**) PL spectra and (**b**) UV-visible DRS spectra of TiO_2_ (P25) and metal ion-doped TiO_2_ photocatalysts.

Figure 6a shows the photoluminescence (PL) spectra of the photocatalysts. The wavelength of the peaks in the PL spectrum of each photocatalyst was 510 nm for NFT, 515 nm for NNT, and 583 nm for NAT. Among the peak spectra of each photocatalyst, the NAT peak appeared at the longest light wavelength (583 nm). The peak spectra of NFT and NNT appeared around 510 nm, which is shorter than that of NFT. However, the optical intensity of NNT was much greater than that of the other photocatalysts. Figure 6b presents the UV-visible light diffuse reflectance spectroscopy (DRS) results of TiO_2_ (P25) and TiO_2_ doped with metal ions and nitrogen. The DRS spectra of TiO_2_ doped with metal ions were shifted to the visible light region compared to TiO_2_ (P25). The DRS spectrum of NNT was shifted the most to the visible light region and was found to absorb visible light over 600 nm. The DRS spectra of the TiO_2_-doped metal ions with nitrogen shifted to the visible area in the order of NNT > NAT > NFT. The bandgap energy of each photocatalyst was obtained by adopting the Kübelka–Münk method [41] from the DRS spectra. The bandgap energy of NNT, NAT, and NFT were ca. 2.4 eV, 2.7 eV, 2.9 eV, respectively. NNT has the narrowest bandgap, so it was judged that it could easily cause a photochemical reaction even in visible light.

### 2.2. Photocatalytic Activities under Visible Light LED Irradiation of N- and Metal Ions-Doped TiO_2_

**Figure 7:**
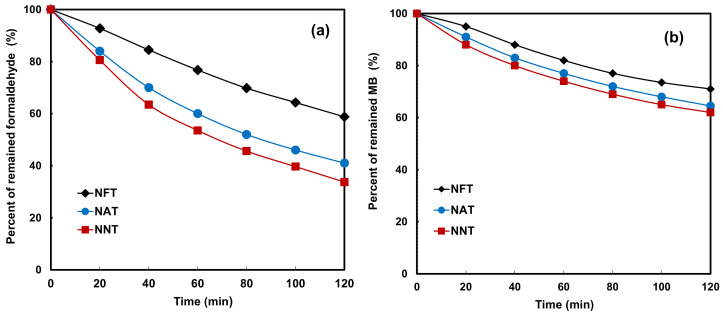
Photocatalytic decomposition of (**a**) formaldehyde and (**b**) methylene blue on NFT, NAT, and NNT.

Figure 7a shows the photolysis activity of the photocatalysts for the photodecomposition of HCHO under LED visible light irradiation. NNT had the highest formaldehyde decomposition activity. NNT and NAT were removed by about 60% after 2 h under visible-light LED illumination. Compared to the two photocatalysts, the degradation activity of NFT was only about 2/3. NAT had narrower bandgap energy than NNT, so activity in visible light was expected to be superior to that of NNT, but the opposite result was shown. Although the bandgap energy of NNT was slightly wider than that of NAT, the PL intensity was much higher, indicating that the photolytic activity was high under the visible light irradiation of the experimental conditions. Figure 7b shows the photolysis activity of each photocatalyst for methylene blue under LED visible light irradiation. In this result, also, the methylene blue decomposition activity of NNTs was the highest. The bandgap of NAT was narrower than that of NNT, but the photocatalytic degradation activity was not higher than that of NNT. This result also seems to be because the optical intensity of NNT is much larger than that of NAT.

**Figure 8:**
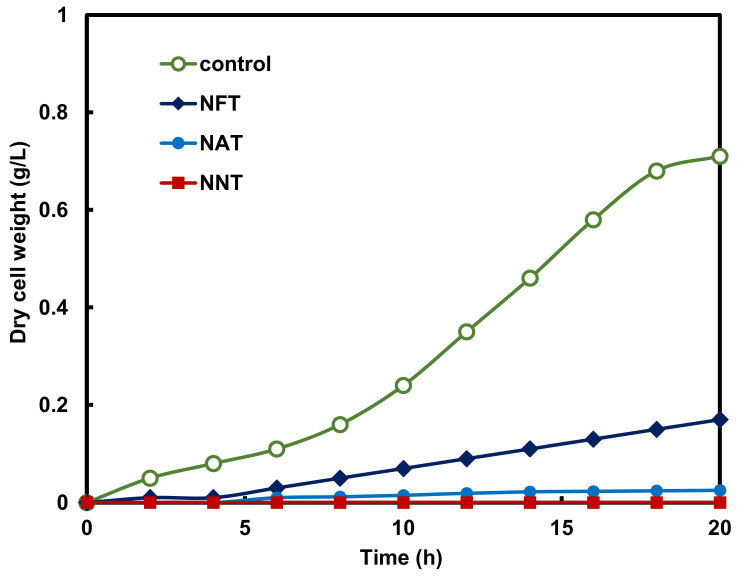
Variation of *Pseudomonas aeruginosa* concentration in the photolysis of various metal ion-doped TiO_2_ photocatalysts under visible light irradiation.

**Figure 9:**
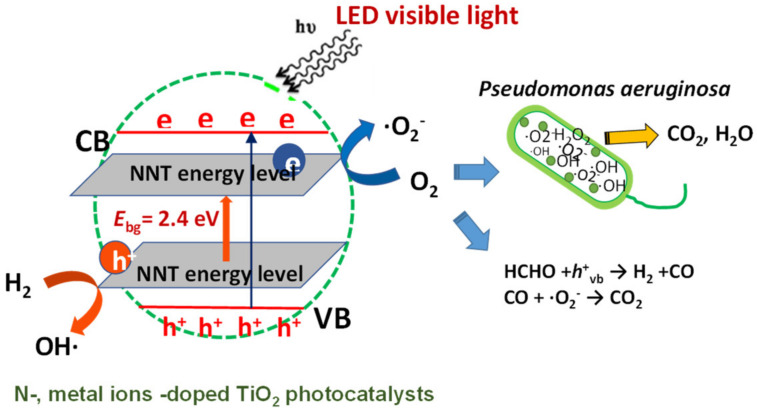
The bandgap change of the TiO_2_ photocatalyst doped with metal ions and nitrogen and the reaction mechanism for organic matter and pathogenic bacteria.

Figure 8 shows the inhibitory effect of photocatalysts on the proliferation of *Pseudomonas aeruginosa* under visible light irradiation. In the *Pseudomonas aeruginosa* culture medium without the photocatalyst injection, the *Pseudomonas aeruginosa* bacteria proliferated significantly, but the *Pseudomonas aeruginosa* culture medium injected with the photocatalyst while irradiating visible light showed an effect of inhibiting the proliferation of *Pseudomonas aeruginosa*. In the *Pseudomonas aeruginosa* culture medium injected with the NNT photocatalyst, *Pseudomonas aeruginosa* did not multiply even after 20 h. This is because the *Pseudomonas aeruginosa* was killed by the activity of the photocatalyst. The excellent degree of the inhibition of the proliferation of *Pseudomonas aeruginosa* by the photocatalyst was shown in the order of NNT > NAT > NFT. It is also judged that the photocatalytic activity is high because the optical intensity of NNT is the highest. Figure 9 shows the bandgap change of the TiO_2_ photocatalyst doped with metal ions and nitrogen and the reaction mechanism for organic matter and pathogenic bacteria.

## 3. Conclusions

In order to produce TiO_2_ that exhibits photocatalytic activity not only in ultraviolet light but also in visible light, several inexpensive metal ions were doped with nitrogen on the surface of TiO_2_ to investigate the photocatalytic properties. Doping with metal ions and nitrogen extended the light absorption region to the visible light region of 620 nm. The PL strength of NNT was much greater than that of NAT and NFT. The bandgap of TiO_2_ doped with metal was significantly reduced compared to commercial TiO_2_ (P25), around 2.0 eV–2.4 eV. The photodegradation activity of NNT was the highest in the formaldehyde decomposition and methylene blue decomposition reactions. In a test evaluating the inhibitory effect on Pseudomonas aeruginosa, the bactericidal effect of NNT was the highest. In visible light, the photocatalytic activity was shown in the order of NNT > NAT > NFT. The NNT had much higher light intensity than NAT or NFT, so it was highly active in visible-light LED irradiation. It was confirmed that the photocatalytic efficiency was the best in TiO_2_ doped with Ni, which is a cheap metal.

## 4. Materials and Methods

### 4.1. Preparation of N- and Metal Ion-Doped TiO_2_ Photocatalysts

TiO_2_ photocatalysts doped with metal ions and nitrogen were prepared as follows. Nanocrystalline TiO_2_ was prepared by adding titanium tetraisopropoxide (TTIP; Dejung, Seoul, Korea, 99.0%) and isopropanol (Duksan, Seoul, Korea, 99%) to distilled water according to the sol-gel method. The TTIP content was hydrolyzed by adding 10 wt% of TiO_2_ to the mass of TiO_2_ at 30 °C for 6 h and then by dropwise adding it to distilled water. A TiO_2_ sol was then synthesized via peptization by injection with 7 mL of nitric acid. Iron(III) nitrate nonahydrate (Fe(NO_3_)_3_·9H_2_O, Duksan, Seoul, Korea), Nickel(II) chloride hexahydrate (Ni(NO_3_)_2_6H_2_O, Wako, Osaka, Japan. 99%), and N/10-silver nitrate (Duksan, Seoul, Korea) reagents were used as the precursors of metal ions, respectively. The content of the metal ion precursor was adjusted to be 1 wt%. The metal ion was doped by stirring at 40 °C for 12 h. (NH_4_)_2_CO_3_ (Samjun, Seoul, Korea, 30%) of 0.02 M was injected into this solution, and the nitrogen was doped while stirring at the same temperature for 6 h to prepare a TiO_2_ photocatalyst sol doped with N and metal ions. This sol was dried at 120 °C in a dryer for 1 day. The dried sample was calcined in a muffle furnace at 500 °C for 10 h to finally prepare TiO_2_ powder doped with N and metal ions. 

### 4.2. Properties of the Photocatalytic Degradation of the Photocatalysts under Visible Light

The photocatalytic degradation of HCHO and MB was performed under visible-light LED irradiation on a TiO_2_ photocatalyst doped with N and metal ions. As a light source, an LED lamp (12 W) kit that combines two 585 nm LED lamps and two 613 nm LED lamps was used. The emission spectrum of the LED lamp was measured in the range of 580 nm to 640 nm. The photocatalytic decomposition experiment of HCHO was performed by illuminating the visible-light LED lamp inside an experimental box that blocked the inflow of external air. The HCHO solution (Duksan, Seoul, Korea, EP, 40%) was vaporized in a vaporizer maintained at 70 °C and the generated gaseous HCHO was introduced into the reaction box. The airflow inside the experimental box was circulated using a fan. For the reaction, 1 g of the photocatalyst was applied. The initial concentration of HCHO and the concentration during the reaction were measured using a gas chromatograph (GC, Younglin, M600D, Anyang, Korea). The MB decomposition activity evaluation experiment was performed by injecting 100 mL of MB solution and 0.5 g of photocatalyst powder into the reactor. An LED lamp was illuminated while the reactant was stirred with a photocatalyst. The change in the concentration of MB was measured using a UV-visible spectrometer (Specgen, Tech Inc., New York, USA).

### 4.3. Inhibition of Pseudomonas aeruginosa Proliferation by Photolysis under Visible Light

The *Pseudomonas aeruginosa* (KCTC 2004) was purchased from the Korean Collection of Type Cultures (KCTC). The nutrient solution used for the photocatalytic growth inhibition test of pathogenic bacteria consisted of peptone (5 g), agar (15 g), beef extract (5 g), and distilled water (1 L). *Pseudomonas aeruginosa* (1 × 10^8^ CFU/mL) was suspended in each nutrient solution (100 mL), and 0.5 g of each photocatalyst and the control group not injected with the photocatalyst were injected thereto. The reactor containing the culture medium and photocatalyst was continuously shaken using a shaker, maintaining 35 °C while illuminated with visible LED light. Cell growth was determined by the dry cell weight (DCW) method. The concentration of *Pseudomonas aeruginosa* was measured at 600 nm using a UV spectrometer (Specgen, Tech Inc., New York, USA) by collecting the culture medium every hour. The cell concentration was determined from a calibration curve of DCW versus absorbance at 600 nm.

### 4.4. Investigation of Physicochemical Properties of Photocatalysts

The crystallinity and structure of TiO_2_ photocatalysts doped with N and metal ions were determined by XRD using a high-resolution XRD system (Rigaku, D/Max Ultima III, Tokyo, Japan). The shapes and microstructures of the photocatalysts were observed by an SEM instrument (Hitachi, S-4850/EX-400, Tokyo, Japan). The TEM images of the photocatalysts were measured using a TEM instrument (JEOL, JEM-2100F). The N_2_ isotherms of the photocatalyst were probed using a volumetric adsorption apparatus (MSI, Nanoporosity PQ, Gwangju, Korea) at −197 °C. The samples were pre-treated at 150 °C for 2 h and exposed to N_2_ gas. The surface area was determined by applicating the BET theory [40]. The binding state of the components of the photocatalyst was investigated with the XPS system (VG Co., MultiLab 2000, East Grinstead, UK). The PL of the photocatalyst was analyzed using a PL spectrometer (Acton Research Co., Spectrometer ioinsgraph 5000i, Massachusetts, USA). The HeCd laser was used for excitation at 325 nm.

## Data Availability

Not applicable.
